# An Empirical Evaluation of Convolutional Networks for Malaria Diagnosis

**DOI:** 10.3390/jimaging8030066

**Published:** 2022-03-07

**Authors:** Andrea Loddo, Corrado Fadda, Cecilia Di Ruberto

**Affiliations:** Department of Mathematics and Computer Science, University of Cagliari, Via Ospedale 72, 09124 Cagliari, Italy; corradofadda1996@gmail.com (C.F.); dirubert@unica.it (C.D.R.)

**Keywords:** computer vision, deep learning, image processing, malaria parasites detection, malaria parasites classification

## Abstract

Malaria is a globally widespread disease caused by parasitic protozoa transmitted to humans by infected female mosquitoes of Anopheles. It is caused in humans only by the parasite Plasmodium, further classified into four different species. Identifying malaria parasites is possible by analysing digital microscopic blood smears, which is tedious, time-consuming and error prone. So, automation of the process has assumed great importance as it helps the laborious manual process of review and diagnosis. This work focuses on deep learning-based models, by comparing off-the-shelf architectures for classifying healthy and parasite-affected cells, by investigating the four-class classification on the Plasmodium falciparum stages of life and, finally, by evaluating the robustness of the models with cross-dataset experiments on two different datasets. The main contributions to the research in this field can be resumed as follows: (i) comparing off-the-shelf architectures in the task of classifying healthy and parasite-affected cells, (ii) investigating the four-class classification on the *P. falciparum* stages of life and (iii) evaluating the robustness of the models with cross-dataset experiments. Eleven well-known convolutional neural networks on two public datasets have been exploited. The results show that the networks have great accuracy in binary classification, even though they lack few samples per class. Moreover, the cross-dataset experiments exhibit the need for some further regulations. In particular, ResNet-18 achieved up to 97.68% accuracy in the binary classification, while DenseNet-201 reached 99.40% accuracy on the multiclass classification. The cross-dataset experiments exhibit the limitations of deep learning approaches in such a scenario, even though combining the two datasets permitted DenseNet-201 to reach 97.45% accuracy. Naturally, this needs further investigation to improve the robustness. In general, DenseNet-201 seems to offer the most stable and robust performance, offering as a crucial candidate to further developments and modifications. Moreover, the mobile-oriented architectures showed promising and satisfactory performance in the classification of malaria parasites. The obtained results enable extensive improvements, specifically oriented to the application of object detectors for type and stage of life recognition, even in mobile environments.

## 1. Introduction

Malaria is a globally widespread disease caused by parasitic protozoa transmitted to humans by infected female mosquitoes of Anopheles. In 2019, there were an estimated 229 million malaria cases worldwide, with an estimated 409,000 deaths due to malaria. Of them, 94% of malaria cases and deaths occurred in Africa [1]. In this context, children under five years of age are the most vulnerable group accounting for 67% (274,000) of all malaria deaths worldwide. Parasites of the genus Plasmodium (P.) cause malaria in humans by attacking red blood cells (RBCs). They spread to people through the bites of infected female Anopheles mosquitoes, called “malaria vectors”. Five species of parasites cause malaria in humans: *P. falciparum*, *P. vivax*, *P. ovale*, *P. malariae* and *P. knowlesi*. *P. falciparum* and *P. vivax* are the two posing the most significant threat [1,2]. The former is most prevalent in Africa, while *P. vivax* is predominant in the Americas. Malaria plasmids within the human host have the following life stages: ring, trophozoite, schizont and gametocyte. The World Health Organization (WHO) defines human malaria as a preventable and treatable disease if diagnosed promptly. Still, the diagnosis must be made promptly, as the worsening illness can lead to disseminated intravascular thrombosis, tissue necrosis and spleen hypertrophy [1,3,4,5].

Blood cell analysis using peripheral blood slides under a light microscope is considered the gold standard for the detection of leukaemia [6,7,8,9], blood cell counting [10,11,12,13,14] or the diagnosis of malaria [15,16,17]. Manual microscopic examination of peripheral blood smears (PBS) for malaria diagnosis has advantages such as high sensitivity and specificity compared to other methods. However, it requires about 15 minutes for microscopic examination of a single blood sample [18], and the quality of the diagnosis depends solely on the experience and knowledge of the microscopist. It is common for the microscopist to work in isolation without a rigorous system to ensure the quality of the diagnosis. In addition, the images analysed may be subject to variations in illumination and staining that can affect the results. In general, the manual process is tedious and time-consuming, and decisions dictated by misdiagnosis lead to unnecessary use of drugs and exposure to their side-effects or severe disease progression [19,20].

This work investigates the classification of malaria parasites using transfer learning (TL) to distinguish healthy and parasite-affected cells and classify the four *P. falciparum* stages of life. Moreover, the robustness of the models has been evaluated with cross-dataset experiments on two very different public datasets.

In this paper, transfer learning will be introduced by explaining how it works and discussing the pretrained networks selected to perform the comparative tests. The experiments are divided into (i) binary, (ii) multiclass and (iii) cross-domain classification. In the latter, networks trained on datasets from different domains were used to see if this improves accuracy over results obtained in a single domain.

The rest of the manuscript is organised as follows. Section 2 presents the literature on computer-aided diagnostic (CAD) systems for malaria analysis. Section 3 illustrates the datasets, methods and experimental setup. The results are presented and discussed in Section 4 and, finally, in Section 5, the findings and directions for future works are drawn.

## 2. Related Work

Several solutions for the automatic detection of malaria parasites have been developed in recent years. They aim to reduce the problems of manual analysis depicted in Section 1 and provide a more robust and standardised interpretation of blood samples while reducing the costs of diagnosis [15,21,22], mainly represented by CAD systems. They can be based on the combination of image processing and traditional machine learning techniques [23,24,25], and also deep learning approaches [16,26,27,28], especially after the proposal of AlexNet’s convolutional neural network (CNN) [29].

Since malaria parasites always affect the RBCs, any automatic malaria detection needs to analyse the erythrocytes to discover if they are infected or not by the parasite and, further, to find the stage of life or the type.

Among the more recent and classical solutions not employing CNNs, Somasekar et al. [23] and Rode et al. [25] proposed two malaria parasite segmentation methods. The first one used fuzzy clustering and connected component labelling followed by minimum perimeter polygon to segment parasite-infected erythrocytes and detect malaria, while the second one is based on image filtering and saturation separation followed by triangles thresholding.

Regarding the CNN-based approaches, Liang et al. [26] proposed a novel model for the classification of single cells as infected or uninfected, while Rajaraman et al. [27] studied the accuracy of CNN models, starting from pretrained networks, and proposed a novel architecture trained on a dataset available from the National Institutes of Health (NIH). They found that some pre-existing networks, by means of TL, can be more efficient than networks designed ad hoc. In particular, ResNet-50 obtained the best performance. Subsequently, they further improved through an ensemble of CNNs [28]. Rahman et al. [30] also exploited TL strategies using both natural and medical images and performed an extensive test of some off-the-shelf CNNs to realise a binary classification.

Some other techniques not explored in this work are based on the combination of CNN-extracted features and handcrafted ones [31,32,33] or the direct use of object detectors [34]. For example, Kudisthalert et al. [33] proposed a malaria parasite detection system, based on the combination of handcrafted and deep features, extracted from pretrained AlexNet. Abdurahman et al. [34] realised a modified version of the YOLOV4 detector. Moreover, they generated new anchor box sizes with a K-means clustering algorithm to exploit the model on small objects.

Finally, a recent focus has been posed on mobile devices, which enable a cheaper and quicker diagnosis in the underdeveloped areas of the world, where more expensive laboratories do not exist. As an example, Bias et al. [24] realised an edge detection technique based on a novel histogram-based analysis, coupled with easily accessible hardware, focused on malaria-infected thin smear images.

The work in [30] is the most similar to the approach here proposed. In particular, they compared different off-the-shelf networks for a binary classification using two datasets, one is the Malaria Parasite Image Database for Image Processing and Analysis (MP-IDB) [35] and another is composed of synthetic and medical images. The task faced, however, is a binary classification. On the entire MP-IDB, they reported 85.18% accuracy with a fine-tuned version of VGG-19.

In summary, the main difference between our work and the state-of-art is that here an extended set of off-the-shelf CNNs on two very different public datasets have been exploited with a dual purpose: detect healthy and unhealthy RBCs and distinguish the various stages of life. Finally, it is the first baseline provided for the stages of life classification on the MP-IDB.

## 3. Materials and Methods

In this section, the datasets, the techniques and the employed experimental setup are described.

### 3.1. Datasets

Two well-known benchmark datasets were used: the National Institutes of Health (NIH) [27], proposed for malaria detection, and MP-IDB [35], a recently proposed dataset for malaria parasite types and stages of life classification.

#### 3.1.1. NIH

**Figure 1:**
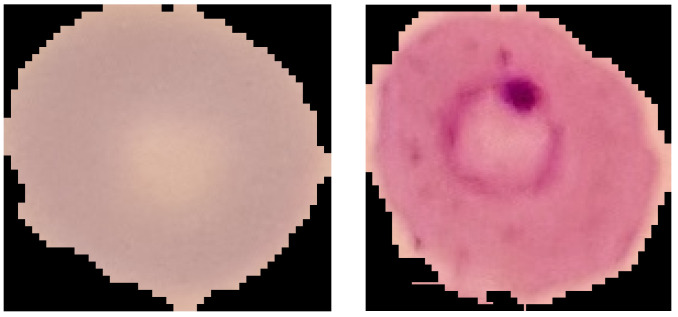
Example of RBCs, healthy (**left**) and sick (**right**), included in the NIH dataset.

The NIH is a public PBS images dataset from healthy individuals and malaria-affected patients. Image acquisition was performed at the Lister Hill National Center for Biomedical Communications from Giemsa-stained blood samples obtained from 150 patients infected with *P. falciparum* and 50 healthy patients. The resulting dataset consists of 27,558 images, uniformly subdivided between infected and healthy cells. The infected cells contain a ring-shaped parasite. Figure 1 shows a healthy and sick RBC extracted from NIH.

#### 3.1.2. MP-IDB

**Figure 2:**
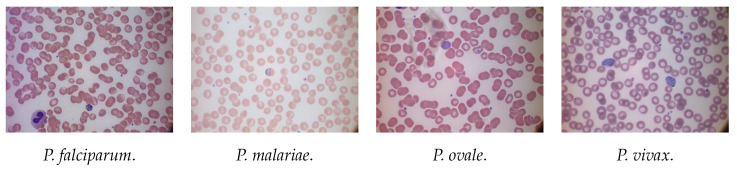
Example of the four types of malaria parasites of MP-IDB.

The MP-IDB consists of four malaria parasite species, *P. falciparum*, *P. malariae*, *P. ovale* and *P. vivax*, represented by 122, 37, 29 and 46 images, respectively, for a total amount of 229. The images have been acquired with 2592×1944 resolution and 24-bit colour depth. Moreover, every species contains four distinct life stages: ring, trophozoite, schizont and gametocyte. Figure 2 shows four examples of the types of malaria parasites included in MP-IDB.

### 3.2. Classification Pipeline

#### 3.2.1. Deep Learning

In this work, deep learning approaches have been used as classifiers. In particular, eleven different off-the-shelf CNN architectures have been evaluated. All of them have been pretrained on the well-known natural image dataset ImageNet [36], and then adapted to this medical image task, as proposed in [37], following a transfer learning and fine-tuning procedure.

AlexNet [29] and VGG-16 [38] are pretty simple and similar architectures, composed of 8 and 16 layers, respectively. Nevertheless, they are widely used for transfer learning and fine-tuning [37], as they have gained popularity for their excellent performance in many classification tasks [29]. GoogLeNet [39] and Inceptionv3 [40] are both based on the inception layer; in fact, Inceptionv3 is a variant of GoogLeNet, using 140 levels, 40 more than GoogLeNet. The 3 ResNet architectures have 18, 50, 101 layers for ResNet-18, ResNet-50 and ResNet-101, respectively, based on residual learning. They are easier to optimise even when the depth increases considerably [41]. The building block of ResNet inspired DenseNet-201. It deals with the vanishing-gradient problem by introducing a connectivity pattern between layers. It is comprised of multiple densely connected layers, and their outputs are connected to all the successors in a dense block [42]. ShuffleNet [43], SqueezeNet [44] and MobileNetV2 [45] are lighter networks. In particular, the last two are oriented to real-time executions and mobile device usage.

Regarding the transfer learning strategy, the approach used in [37] was followed. All CNN layers were retained except for the last fully connected one. It was replaced with a new layer, initialised and set up to accommodate new categories according to the classification strategy exploited (two classes in the binary one and four in the multiclass one).

#### 3.2.2. Image Preprocessing

All the images have a uniform background, even if different according to the dataset. Indeed, NIH contains only single-cell images surrounded by a black background, while the images of MP-IDB have several blood components with their actual plasma background. Both datasets are organised into classes, healthy or sick, for NIH and the four malaria types for MP-IDB. As it can be seen from Figure 1 and Figure 2, there are many colour variations between the images of both datasets. This condition is undoubtedly due to the different acquisition conditions and from the status of the blood smear [35]. Therefore, it was considered appropriate to apply a preprocessing step to realise a colour balance. Furthermore, for MP-IDB, we created a single image for each parasite from the full-size images. The preprocessing step was designed mainly to adjust the colour components of the images and applied to all the RGB channels, using a colour-balancing technique, through Equation (1), where $C_{out}$ is the processed component, $C_{in}$ is the component to be processed, $m_{im}$ is the average of the average intensities of the three channels and, finally, $m_{c}$ is the average intensity of the component to be processed. This procedure was carried out on all three channels of the RGB image.

(1) $$C_{out}=C_{in}\frac{m_{im}}{m_{c}}$$

**Figure 3:**
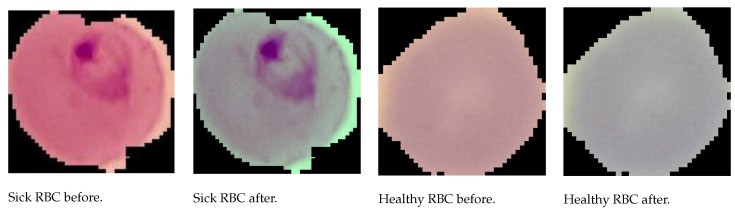
Examples of NIH images, before and after preprocessing step.

**Figure 4:**
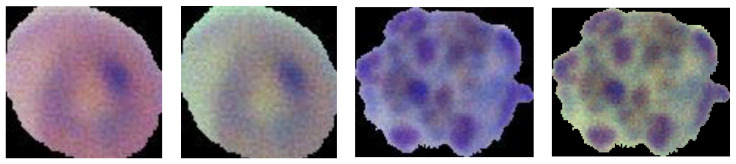
Examples of MP-IDB-FC images, before and after preprocessing step.

As far as MP-IDB is concerned, the work was also oriented to generate single-cell images from the full-size and, more specifically, the Falciparum class. From now on, we refer to this dataset as MP-IDB-Falciparum-Crops (MP-IDB-FC). MP-IDB contains 104 patient-level images with the corresponding ground truths of the infected cells. As the desired classification was at the cellular level, individual cells had to be extracted from the images and labelled using the class references in the file names. The Falciparum class presents the following image distribution per stage: 1230 rings, 18 schizonts, 42 trophozoites and 7 gametocytes. In Figure 3 and Figure 4, the preprocessing step results are shown.

Finally, to overcome the class imbalance issue in the dataset produced, a further augmentation step was applied and described below.

#### 3.2.3. Data Augmentation

Different data augmentation techniques, such as flipping, shifting and rotating, are used to overcome the problem of a limited dataset and to produce further copies of the original image to give the algorithm more generalisation capability and reduce the error rate [29,46,47,48].

MP-IDB-FC presented an unbalanced distribution of images per class; therefore, we proposed an offline data augmentation to oversample the underrepresented classes. The applied geometric transformations are random rotations between −90° and 90°, random translations between −10 and 10 pixels on the X-axis and Y-axis, and 50% reflection around the X-axis and Y-axis.

#### 3.2.4. Experimental Setup

As previously introduced, the images to be classified are microscopic blood smear images. More specifically, NIH contains single-cell images representing healthy or ring stage malaria parasite-affected RBC, while in the case of MP-IDB-FC, the images represent a single cell containing one of the four stages of *P. falciparum*.

In the case of NIH, the dataset was divided into three parts, one for the training (80%), one for the validation (10%) and 10% for testing. A stratified sampling procedure to keep the splits balanced was used, as NIH classes are well balanced.

As for MP-IDB, in order to consider the class imbalance and to have a sufficient number of samples for the training process while preserving a sufficient number of samples for performance evaluation, the dataset was split first into two parts, namely training and testing set, with 80 and 20% of images, respectively. A total of 10% of the training set was used for validation due to the small number of images. Naturally, the subdivision of the sets was carried out before oversampling to avoid that train or validation images fell into the test set, thus compromising the results. The subdivision was made to ensure that 20% of each class made up the test set, thus avoiding the circumstance where no class images with few elements were in the test set. Oversampling and undersampling were then adopted to increase the number of images to 100, 200 or 300 per class in the training set. Moreover, the splits were not created randomly but by taking the images in lexicographic order from each class to further ease reproducibility. All the experiments have been conducted on a single machine with the following configuration: Intel(R) Core(TM) i9-8950HK @ 2.90 GHz CPU with 32 GB RAM and NVIDIA GTX1050 Ti 4GB GPU. Finally, no randomisation strategy has been applied to make all the experiments on both datasets reproducible.

This work represents a baseline for further investigation and searching for the best architecture for our purpose. The selected CNNs have been employed without any modification to their architecture. In particular, after empirical evaluation, Adam algorithm was adopted, which performed better than the other solvers. In addition, the maximum number of epochs was set to 10 due to the number of images.

As mentioned in Section 3.2.1, a fine-tuning process was applied on all the CNN architectures exploited on both datasets. The hyperparameters defined in Table 1 were used for all networks to evaluate potential performance variations. Furthermore, the regularisation factor L_2_ was set to avoid a possible overfitting during the training phase.

## 4. Experimental Results

Three different experiments were conducted, according to the classification purpose:

- Binary classification on the NIH dataset (healthy vs. sick);
- Multiclass classification on the MP-IDB-FC dataset (four stages of life);
- Multiclass cross-dataset classification on both datasets.

The results obtained in the analysis of each experiment were performed using the confusion matrix. The confusion matrix metric used in this study is *Accuracy*. The formula of this metric is given in Equation (2). The variables used in the equation are True Positive (*TP*), False Positive (*FP*), True Negative (*TN*), and False Negative (*FN*), parameters of the confusion matrix used to calculate the metrics [49,50].

(2) $$Accuracy=\frac{TP+TN}{TP+TF+FP+FN}$$

### 4.1. Binary Classification Performance on NIH

To determine the training options to use, test trials were carried out. From them, it was mainly verified that:

- Extending the training phase beyond ten epochs did not improve accuracy, as the network stored individual image features rather than class features, and overfitting compromised the results;
- The ideal learning rate was 1 × 10^−4^. The accuracy increased too slowly for smaller values, and for larger ones, it did not converge to a specific value;
- Empirically, Adam was found as the best solver.

Table 2 shows that almost all the networks have an accuracy value close to the average. The standard deviation of the collected data is solely 0.16%. This aspect could be because the dataset used has many valuable images for training the network. In particular, ResNet-18 recorded the highest accuracy value, confirming the high performance expressed in [27]. MobileNetV2, SqueezeNet and ShuffleNet recorded average values, which is an important result since they are networks designed for mobile use.

### 4.2. Multiclass Classification Performance on MP-IDB-FC

The multiclass classification on MP-IDB-FC was designed to determine the life stage of the parasite: ring phase, adult trophozoite, schizont and gametocyte.

Like the binary classification, comparative tests with the same training set, validation set and test set were carried out to determine which networks perform best and allow comparison between them. We created three datasets with 100, 200 or 300 images per class in the training set. We refer to these sets as D1, D2 and D3, respectively. They were constructed by oversampling with augmentation of the remaining classes.

Table 3 shows the performance in this experiment. Each test was cross-validated five times; then, we reported the mean accuracy and standard deviation considering each of the five folds. The most notable result is that the average performance from D2 to D3 worsens. However, DenseNet-201 and GoogLeNet are the only networks to benefit from increasing the dimensionality of the training set.

### 4.3. Cross-Dataset Classification Evaluation

The cross-domain classification was carried out to evaluate the CNNs robustness.

#### 4.3.1. MP-IDB-FC Classification with NIH Models

Firstly, two different multiclass classifications were realised on MP-IDB-FC by:

- Training on NIH and testing on MP-IDB-FC (Exp1);
- Training on NIH + fine-tuning on MP-IDB and testing on MP-IDB-FC (Exp2).

When used for training or fine-tuning, the split on MP-IDB-FC was 50% for training (with 10% for validation) and 50% for testing. Every test was evaluated with five-fold cross-validation. Therefore, we report the average accuracy of the five folds and the standard deviation. This cross-domain experiment tested whether the networks trained on the NIH dataset could be employed on the MP-IDB-FC dataset. It is helpful to point out that the significant difference is that, on the one hand, MP-IDB-FC has parasite crops and not healthy RBCs and, on the other hand, NIH contains only ring-stage parasites. For this reason, the objective of Exp1 was to discriminate between rings and the remaining stages of life, while Exp2 aimed to expand the knowledge of the NIH pretrained models with new information on the stages of life.

The results depicted in Table 4 show that when the target domain differs excessively from the source domain, it is hard to directly apply the models trained with NIH to MP-IDB-FC (Exp1), even if the task seemed feasible, as ring-stage parasites were contained in both datasets. Conversely, Exp2 shows that using the CNNs first trained on NIH and then fine-tuned on MP-IDB-FC led to an improvement in average accuracy. The information about healthy RBCs provided with NIH training does not affect the overall result. In addition, the standard deviation is under 4% for all the networks, leading to satisfactory performance stability.

#### 4.3.2. *P. vivax* Classification Using *P. falciparum* Data

The last experiment aimed to investigate the possibility of classifying the stages of life of *P. vivax* using the information on *P. falciparum*. So, a different dataset was created and composed of the crops of *P. vivax* parasites, referred to as MP-IDB-VC. Even in this case, three different evaluations were conducted by:

- Training on MP-IDB-VC and testing on MP-IDB-FC (Exp3);
- Training on MP-IDB-VC and testing on MP-IDB-VC (Exp4);
- Training on MP-IDB-FC, fine-tuning and testing on MP-IDB-VC (Exp5).

Trophozoites, schizonts and gametocytes greatly vary between the two types, while the ring stages are pretty similar.

As it can be seen from Table 5, the classification of *P. falciparum* stages of life employing models trained *P. vivax* produced dreadfully low results due to the differences between all the stages except rings. On the other hand, using same-domain models (Exp4) had satisfactory results. Exp5 demonstrates that the fine-tuning strategy on the models pretrained on *P. falciparum* improved the accuracy, as already happened in Section 4.3.1 Exp2. In this task, DenseNet-201 provided the best performance, being the only CNN to overcome 85% and outperforming the average of 10% in both cases.

## 5. Conclusions

The results obtained in this work support the importance of deep learning in haematology. This work aimed to demonstrate that pretrained off-the-shelf networks can offer high accuracy for diagnosing malaria utilising transfer learning however showing several limitations of this approach. Several comparative tests were developed using a selection of pretrained networks differentiated by size, depth and the number of parameters. In particular, using the NIH dataset, it is possible to distinguish a healthy from an infected erythrocyte with an accuracy of over 97%. Small networks such as SqueezeNet and ShuffleNet performed well, consolidating a possible development of software for malaria diagnosis in small devices such as smartphones. On the other hand, MP-IDB has highlighted some critical issues: deep learning is not very effective when the dataset used for training is unbalanced. Some classes of parasites in the dataset have a small number of images. Nevertheless, the oversampling, augmentation and preprocessing methods still allowed us to exceed 90% accuracy on the test set for distinguishing the four life stages of the *P. falciparum* parasite. Finally, the cross-domain experiments have highlighted some critical points in classifying data from heterogeneous domains. It was counterproductive to apply the models trained with NIH to MP-IDB-FC, but the use of the CNNs firstly trained on NIH and fine-tuned on MP-IDB-FC led to an improvement in average accuracy. This aspect also applies to using the *P. vivax* dataset as the target domain, as most of the classes deviate too much from the corresponding *P. falciparum* classes. However, using both types of parasites as source domains produced better results than training on *P. vivax* only. In general, the extensive experimentation has highlighted how DenseNet-201 offers the most stable and robust performance, offering itself as a crucial candidate for further developments and modifications.

Among the possible developments of this work, we aim to propose a framework able to detect malaria parasites from blood smear images and classify different species of parasites and different stages of life, mainly focusing on high variation data. We also plan to use domain adaptation algorithms to improve cross-domain performance.

## Figures and Tables

**Table 1:** Hyperparameters settings for CNNs fine-tuning.

Params	Value
Solver	Adam
Max Epochs	10
Mini Batch Size	32
Initial Learn Rate	1 × 10^−4^
Learn Rate Drop Period	10
Learn Rate Drop Factor	0.1
L_2_ Regularisation	0.1

**Table 2:** Accuracy and training times of CNNs on NIH with preprocessing step.

Network	Accuracy (%)	Time (min)
AlexNet	97.35	10
DenseNet-201	95.86	5145
ResNet-18	97.68	21
ResNet-50	97.61	82
ResNet-101	97.24	391
GoogLeNet	96.73	111
ShuffleNet	97.39	33
SqueezeNet	97.21	16
MobileNetV2	97.31	210
Inceptionv3	96.70	151
VGG-16	97.31	322
Avg.	97.25	–

**Table 3:** Average accuracy and standard deviation computed on the same test set, after training with 100, 200 and 300 images (D1, D2, D3, respectively).

	Accuracy ± Standard Deviation (%)		
Network	D1	D2	D3
AlexNet	83.59±12.90	89.74±2.56	89.23±2.15
DenseNet-201	94.15±3.34	95.74±2.56	99.40±0.40
ResNet-18	90.77±6.88	95.38±2.81	89.74±5.44
ResNet-50	91.79±4.50	94.36±3.80	86.15±3.89
ResNet-101	94.87±2.81	92.31±2.56	92.31±3.89
GoogLeNet	92.82±3.34	92.31±2.56	93.85±1.40
ShuffleNet	92.82±2.81	91.28±3.89	89.74±4.10
SqueezeNet	90.26±6.88	88.72±7.82	88.21±8.02
MobileNetV2	87.18±3.34	84.62±5.90	79.49±8.42
Inceptionv3	93.85±3.80	92.31±2.56	84.62±2.56
VGG-16	93.85±3.34	92.31±4.18	87.18±4.50
Avg.	91.42	91.64	88.75

**Table 4:** Cross-dataset experiments for multiclass classification on MP-IDB-FC.

Network	Exp1 (%)	Exp2 (%)
AlexNet	42.71	88.21±3.44
DenseNet-201	49.73	97.45±1.40
ResNet-18	68.23	94.87±2.56
ResNet-50	68.23	92.31±3.63
ResNet-101	69.10	94.87±2.29
GoogLeNet	68.23	89.23±3.80
ShuffleNet	32.23	95.90±1.40
SqueezeNet	68.23	87.18±3.14
MobileNetV2	57.12	89.74±2.29
Inceptionv3	41.84	93.85±3.44
VGG-16	53.59	94.87±2.81
Avg.	60.61	92.59

**Table 5:** Cross-dataset experiments for multiclass classification on MP-IDB-VC.

Network	Exp3 (%)	Exp4 (%)	Exp5 (%)
AlexNet	30.16	77.14±9.31	71.43±11.29
DenseNet-201	72.94	88.89±5.56	87.10±1.15
ResNet-18	30.16	82.86±3.91	82.86±6.93
ResNet-50	55.56	78.57±8.74	78.57±5.05
ResNet-101	57.14	82.86±3.91	81.43±3.91
GoogLeNet	28.57	68.57±3.91	74.28±3.91
ShuffleNet	58.73	82.86±3.91	82.86±3.91
SqueezeNet	31.75	80.00±5.98	80.00±5.98
MobileNetV2	34.92	67.14±3.91	74.28±6.39
Inceptionv3	57.14	78.57±5.05	78.57±5.05
VGG-16	49.21	77.14±3.19	81.43±3.91
Avg.	46.02	78.60	79.35

## Data Availability

All the material used and developed for this work is available at the following GitHub repository. All the models trained for the different experimentations can be found at this repository.

## Abbreviations

The following abbreviations are used in this manuscript:

Plasmodium	*P.*
RBC	Red Blood Cell
PBS	Peripheral Blood Smears
CAD	Computer-Aided Diagnostic
CNN	Convolutional Neural Network
TL	Transfer Learning
MP-IDB	Malaria Parasite Image Database for Image Processing and Analysis
NIH	National Institutes of Health
